# Do Self-adhesive Resin Composites Release More Monomers? A Comparative High-performance Liquid Chromatographic Analysis

**DOI:** 10.3290/j.jad.b3240709

**Published:** 2022-08-18

**Authors:** Loulwa M. Al-Saud, Alhassan H. Aodah, Omar A. Abu Asab

**Affiliations:** a Assistant Professor and Consultant of Operative Dentistry, Department of Restorative Dental Sciences, College of Dentistry, King Saud University, Riyadh, Saudi Arabia. Research idea, hypothesis, experimental design, specimen preparation, wrote the manuscript, performed statistical analysis, discussed the results, proofread the manuscript, commented on the manuscript at all stages.; b Assistant Professor of Pharmaceutics and Industrial Pharmacy, Deputy Executive Director, Life Science and Environment Research Institute, King Abdulaziz City for Science and Technology (KACST), Riyadh, Saudi Arabia. Contributed to experimental design, monitored HPLC experiments, contributed to methodology writing, critically proofread the manuscript.; c Pharmaceutical Analysis Laboratory Senior Supervisor, King Abdulaziz City for Science and Technology (KACST), Riyadh, Saudi Arabia. Contributed to experimental design, performed HPLC analysis, contributed to methodology writing, critically proofread the manuscript.

**Keywords:** self-adhesive resin composite, HPLC, monomer elution, liquid chromatography, giomers, nanohybrid.

## Abstract

**Purpose:**

To comparatively evaluate the elution of residual monomers (bis-GMA, bis-EMA, TEG-DMA, and HEMA) from two self-adhesive flowable resin composites, a giomer, and a nano-flowable resin composite over five different time intervals, using high-performance liquid chromatography (HPLC).

**Materials and Methods:**

Four flowable resin composites were investigated (Vertise Flow, Constic, Beautifil Flow Plus F03, and Filtek Z350 XT). Immediately after polymerization, each sample was immersed in 75% ethanol/water solution and stored in amber-colored bottles at room temperature. HPLC analysis was performed at predefined time intervals: 1 h, 24 h, 4 days, 8 days, and 16 days. The extraction solution was changed after each analysis. Data were analyzed with repeated-measures ANOVA and one-way ANOVA with Tukey’s post-hoc test at p < 0.05.

**Results:**

The highest mean concentration of residual monomers was eluted from Beautifil, followed by Filtek, and both were significantly higher (p < 0.05) than the mean concentration of eluates from self-adhesive resin composites (Vertise Flow and Constic). Vertise Flow released significantly higher concentrations of HEMA than all the other tested materials. At 1 h post-immersion, 52.2% of monomers were eluted, and continued to elute at a reduced rate throughout the study duration. TEG-DMA was the fastest monomer to leach out, while bis-GMA exhibited significantly higher total mean concentration. The elution rate was significantly dependent on the molecular weight of the eluted monomers.

**Conclusion:**

No specific elution behavior can be attributed to self-adhesive RBCs. Elution of residual monomers is dependent on each material’s composition, resin matrix characteristics, and the monomer’s molecular weight.

Since their introduction in the 1960s, resin-based composites (RBCs) have undergone substantial modifications and improvements of their functional properties, versatility, and handling characteristics.^30,35^ However, despite their continuous improvements and widespread use in daily dental practice, there are still some genuine concerns regarding their longevity, biocompatibility, and higher incidence of associated secondary caries.^11,16,32,35,45^

After restoration placement, free radical polymerization and polymer crosslinking reaction of the RBC are never fully completed, as not all monomers convert to polymers.^54^ A considerable amount of residual unreacted or partly reacted monomers remains in the polymeric matrix; these subsequently leach out from the polymerized restoration into saliva as a result of chemical biodegradation.^3^ The eluted monomers and other substances (eg, oligomers, filler particles, and degradation products) not only interact with oral tissues (eg, causing pulpal and gingival irritation), and oral microbiota (eg, increasing bacterial proliferation) but also impact the material’s behavior, structural stability, and longevity in the oral cavity.^4,15,19,32^ Moreover, several studies have shown that some eluted monomers from RBCs may result in cytotoxic, genotoxic, mutagenic or estrogenic effects, leading to local and systemic allergic reactions.^2,14,18,46^

Therefore, the evaluation of released monomers from polymerized RBCs is considered a valuable estimate of the material’s biocompatibility.^37^ The amount of eluted monomers as well as the time needed for their complete elution from each material are of prime importance.^49,53^

Various factors may influence monomer elution from resin-based composites, such as the characteristics of base monomers (size, chemical structure, molecular weight, hydrophobicity, etc), as well as the type and composition of the resin composite (eg, filler content, resin matrix characteristics).^24,37^

Moreover, the concentration of released monomer and the elution rate are dependent on the type and chemical characteristics of the solvent used (eg, hydrophobicity and swelling capacity).^27^ Different types of hydrophilic, hydrophobic, or mixed extraction media have been used in monomer elution studies, for instance, cell culture medium, distilled water, artificial saliva, alcohol mixtures, and other organic solvents. Higher monomer elution has been reported with the use of organic solvents because they can more easily penetrate and swell the organic matrix.^28,37,54^

High-performance liquid chromatography (HPLC) is the most commonly used analytical technique for elution analysis. Other qualitative and quantitative techniques have been also used to examine the release of unreacted monomers from RBCs (gas chromatography, ultra HPLC, gas chromatography/mass spectrometry, high-temperature GC [HT-GC], and electrospray ionization/mass spectrometry).^5,37,42,54^

In an attempt to manage the technique sensitivity of RBC and simplify its clinical application steps, self-adhesive (SACs) and self-etch resin composites were developed. The acidic monomer (etchant), primer/adhesive agent, and restorative material were combined in a single step, thus considerably reducing the time required for clinical application. Basically, SACs are methacrylate-based materials that incorporate functional acidic amphiphilic monomers (eg, glycerol phosphate dimethacrylate [GPDM]) in addition to HEMA into their organic matrix.^9,10,23,33^

The inclusion of acidic monomers in these materials has raised various concerns regarding their influence on the material’s mechanical and physical properties, dimensional stability, and biocompatibility, both during and after polymerization.

Since their introduction in the late 2000s, significant in-vitro and in-vivo research efforts have been directed at evaluating various functional properties of SACs, their adhesion effectiveness to enamel and dentin, and their clinical performance.^6,10,23,31,33,34,36,44,47^ However, the literature lacks data regarding the extent of residual monomer eluted from these materials and whether they release more monomers compared to other types of RBCs.

Therefore, the aim of this in-vitro study was to comparatively evaluate the extent of monomer elution (bis-GMA, bis-EMA, TEG-DMA, and HEMA) from two self-adhesive flowable resin composites, a giomer, and a nano-flowable resin composite over five specified time intervals (a total of 16 days).

The null hypothesis tested was: After polymerization, there is no significant difference in the extent of monomers eluted from self-adhesive resin composites compared to other tested RBCs.

## MATERIALS AND METHODS

### Materials

Four commercially available flowable resin composites were evaluated in this study: two self-adhesive resin composites (Vertise Flow [Kerr; Orange, CA, USA] and Constic [DMG; Hamburg, Germany]), a giomer (Beautifil Flow Plus F03, Shofu; Kyoto, Japan) and a nanohybrid (Filtek Z350 XT [3M Oral Care; St Paul, MN, USA]). Detailed information on the tested materials is presented in Table 1. The analyzed monomers are presented in Table 2.

**Table 1:** Resin composite materials used in the current study (manufacturers’ information)

Resin composite	Type /shade	Manufacturer	Lot No.	Resin matrix	Inorganic filler	Filler% (w/v)
Vertise Flow (VF)	Self-adhering flowable composite/ A2	Kerr; Orange, CA, USA	7385592	GPDMA, HEMA, bis-GMA, catalysts	Prepolymerized filler, silanated barium glass, nano-sized colloidal SiO_2_, YF_3_ Ytterbium fluoride (YbF_3_)	(70, 48)
Constic (CT)	Self-etching, self-adhesive flowable composite/ A2	DMG; Hamburg, Germany	222128	Bis-GMA, EBADMA, UDMA, HEMA, TEG-DMA, HDMA, MDP	Barium glass filler	(66, 43)
Beautifil flow Plus F03 (low flow) (BF)	Fluoride-releasing nano-hybrid flowable (Giomer)/ A2	Shofu; Kyoto Japan	111887	Bis-GMA, TEG-DMA	Multi-functional glass filler and S-PRG filler based on fluoro-alumino-silicate glass	(66.8, 46.3)
Filtek Z350 XT (FT)	Low-viscosity, light cured, radiopaque nano-filled flowable/A2	3M Oral Care; St Paul, MN, USA	NC14421	Bis-GMA, bis-EMA,TEG-DMA, procrylate resins	Ytterbium trifluoride filler, surface modified 75-nm silica filler, surface-modified aggregated zirconia/silica cluster filler (comprised of 20- nm silica and 4- to 11-nm zirconia particles)	(65, 46)

Bis-GMA: bisphenol A-glycidyl methacrylate; GPDMA: glycerol phosphate dimethacrylate; EBADMA: ethoxylated bisphenol A-dimethacrylate.

**Table 2:** Dental monomers investigated in the current study

Analyte	Name	Function	Molecular formula	Molecular weight (g/mol)	CAS Number*
Bis-GMA	Bisphenol A-glycidyl methacrylate	Monomer	C29H36O8	512.6	1565-94-2
Bis-EMA	Bisphenol A-ethoxylate dimethacrylate	Monomer	C35H48O10	628.7	41637-38-1
TEG-DMA	Triethylene glycol dimethacrylate	Co-Monomer	C14H22O6	286.32	109-16-0
HEMA	2-hydroxyethyl methacrylate	Co-Monomer	C6H10O3	130.14	868-77-9

*Chemical Abstract Service Registry number.

### Specimen Preparation

Disk-shaped specimens (thickness: 2 mm; diameter: 3 mm; total surface area: 33 mm^2^) of the flowable resin composites were prepared using a split Teflon mould (n = 12 per material). The mold was sandwiched between two Mylar strips and microscopic glass slides (1 mm thickness) on each side to prevent the formation of an oxygen inhibition layer and to achieve a smooth surface of specimens, under ambient temperature of 23 ± 1°C and relative humidity of 50 ± 2%.

The specimens were polymerized from the top surface for 20 s with a polywave light-emitting diode curing unit (Bluephase, Ivoclar Vivadent; Schaan, Liechtenstein) under standard curing mode. The light-curing unit had an output irradiance of 1200 mW/cm^2^ and wavelength range of 430–480 nm. The curing distance was standardized at 1 mm by using a 1-mm glass slide. A calibrated radiometer system (Bluephase meter, Ivoclar Vivadent) was used to verify the irradiance before each use.

Immediately after curing, each specimen was gently pushed out of the mold and the excess flash of the material was removed using a sharp blade. Subsequently, each specimen was fully immersed in 2 ml of the extraction media (75 wt% ethanol-water solution) and stored in amber-colored bottles at room temperature. The ratio between the specimen and the extraction solution volume was greater than 1:10 (according to ISO 10993-13 standards).^22^

Ethanol-water samples (2 ml) were collected for HPLC analysis at predefined time intervals: 1 h (T1), 24 h (T2), 4 days (T3), 8 days (T4), and 16 days (T5). The extraction solution was refreshed after each analysis.

### HPLC Analysis

**Fig 1:**
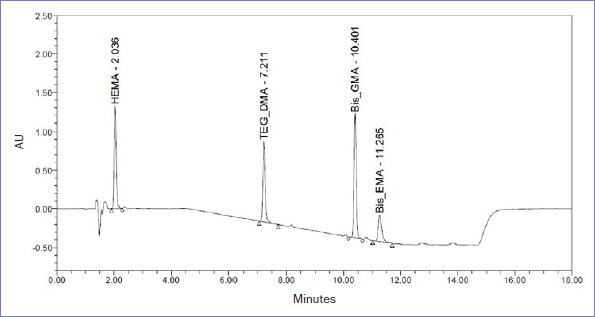
Representative chromatogram for the retention time (minutes) of HPLC peaks of HEMA, TEG-DMA, bis-GMA and bis-EMA under the experimental conditions.

Residual monomer eluates from the specimens were analyzed by an HPLC instrument (J18SM4916A, Waters; Milford, MA, USA) equipped with a µBondapak column (A125, C18, 300 x 3.9 mm x 10µ-C18, Waters). The mobile phase was a mixture of 0.1% formic acid and acetonitrile (HPLC Grade, Honeywell; Offenbach am Main, Germany). The flow rate was 2 ml/min, and the injection volume was 15 µl. UV detection was performed at 205 nm (for monitoring the elution of HEMA, TEG-DMA, bis-GMA and bis-EMA) at the retention times or 2.0 min, 7.2 min, 10.4 min, and 11.3 min. The monomers were identified by comparison of their retention times with those of the reference compounds under the same HPLC conditions. A representative chromatogram of the tested monomers is shown in Fig 1. Analytical standards, which were used for the calibration of the HPLC system to produce stock solutions of 100 µg/ml each, were obtained from Sigma-Aldrich (St Louis, MO, USA). The stock solutions were diluted with 75% ethanol to produce the final calibration solutions: 5, 10, 25, 50 and 100 µg/ml. All chemicals used were of liquid chromatographic grade. The applied analytical technique was adapted from previous studies with the suitable modification needed for the separation of current tested monomers.^5,50^

**Fig 2:**
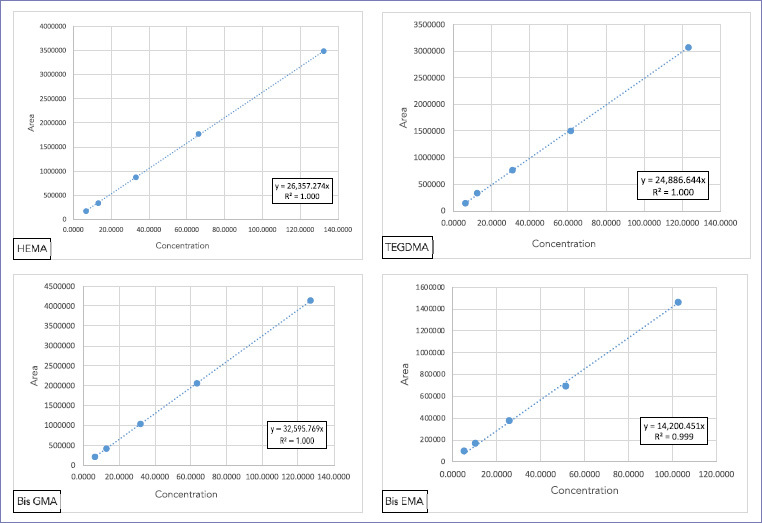
HPLC calibration curves for the monomers HEMA, TEG-DMA, bis-GMA, and bis-EMA.

The peak area for each monomer was determined and plotted against concentration using linear regression analysis (expressed as coefficient of determination r^2^) and used to quantify monomer concentration in the sample solutions. The obtained calibration curves showed high linearity (r^2^= 1; Fig 2).

Specimens were prepared at the College of Dentistry Research Center, KSU, Saudi Arabia (CDRC # FR0578). HPLC analysis was performed at the Pharmaceutical Analysis Laboratory (KACST).

### Statistical Analysis

Preliminary tests of normality (Shapiro-Wilk’s test) and homogeneity of variances (Levene’s test) for equality of variances were performed. The obtained data were analyzed using the following tests at a significance level of p < 0.05: one-way ANOVA with Tukey’s post-hoc test to detect significant differences in a) the concentration of each eluted monomer from different materials; b) the concentration of each eluted monomer at each time interval from different materials; c) linear regression analysis between molecular weight and elution rate of the tested monomer.

Additionally, one-way repeated measures ANOVA was run to detect significant differences in the concentration of eluted monomers from each material at the five pre-defined time intervals. Data were analyzed using SPSS (v.20, IBM; Armonk, NY, USA) and GraphPad Prism (v.9.3.1, (GraphPad Software; San Diego, CA, USA) at a significance level of p < 0.05.

## RESULTS

The highest total mean concentration of residual monomers was released from Beautifil followed by Filtek, both of which released significantly higher amounts of monomers compared to self-adhesive resin composites (Vertise Flow and Constic). Therefore, the first null hypothesis was rejected.

Over the duration of the experiment, the peak elution of the tested monomers was observed at 1 h, which corresponds to 52.2% of the total eluted monomers. The elution continued throughout the experiment duration, albeit with significant gradual reduction in elution rate from all tested materials.

A linear regression was run to understand the effect of monomer’s molecular weight on its elution rate. Regression analysis indicated a significant negative medium correlation between the rate of monomer elution and its molecular weight (r^2^ = 0.526, p = 0.001) (Fig 5).

### HEMA

**Fig 3:**
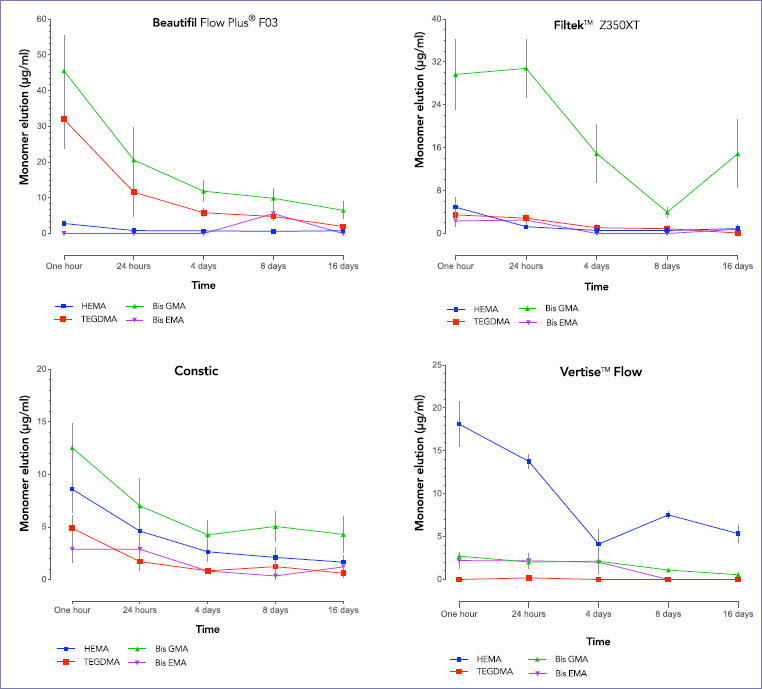
Mean concentration (µg/ml) of eluted monomers from each tested resin composite material at the specified immersion time intervals. Error bars represent standard error of the mean.

A statistically significant difference (p = 0.000) in the eluted HEMA between the tested materials was detected. The mean released HEMA from Vertise Flow (VF) was significantly higher (p = 0.000) than all other materials and corresponded to 59% of the total eluted HEMA (Table 3). HEMA eluted from VF at T1 was statistically higher (p = 0.000) than the released amount at T3, T4, and T5 (Fig 3). Similarly, the amount of HEMA eluted from FT (Filtek) at T1 was statistically significantly greater (p = 0.007) than the released amounts at T2, T3, T4, and T5. At T1, HEMA eluted from BF (Beautifil) and CT (Constic) was significantly higher (p < 0.05) than the elution at T3, T4, and T5.

**Table 3:** Mean (SEM) concentrations in µg/ml of monomers eluted from the tested materials

Monomer	Material				Mean total
	Beautifil	Filtek	Constic	Vertise Flow	
HEMA	1.20 (0.33)	1.68(0.47)	3.93 (0.94)	9.78* (1.17)	4.148 (0.51)
TEG-DMA	11.25* (3.2)	1.70(0.41)	1.86 (0.56)	0.04 (0.03)	3.71 (0.89)
Bis-GMA	18.89* (4.2)	18.87* (3.9)	6.64 (1.3)	1.71 (0.19)	11.53* (1.6)
Bis-EMA	1.12 (0.63)	1.14 (0.38)	1.64 (0.56)	1.28 (0.40)	1.29 (0.24)
Total	32.57* (7.4)	23.39 (4.26)	14.17 (3.16)	12.82 (1.5)	20.69 (2.4)

*Statistically significant (p < 0.05).

### TEG-DMA

Beautifil eluted the highest mean concentration of TEG-DMA of all tested materials (p = 0.000), which corresponds to 75.7% of the total TEG-DMA elution. The peak elution was detected at T1, and it was significantly higher (p = 0.010) than the elution at T3, T4, and T5. Similarly, the eluted TEG-DMA from FT at T1 was statistically higher (p = 0.031) than the elution at T5. Constic elution at T1 was significantly higher (p < 0.05) than the elution at T3, T4, and T5. On the other hand, the elution of TEG-DMA from VF was non-detectable (except at T2).

### Bis-GMA

The mean eluted bis-GMA from BF and FT was significantly higher (p = 0.000) than the elution from self-adhesive materials (CT and VF). Over the 5 different time intervals, the peak elution from BF was detected at T1 and it was significantly higher (p = 0.011) than the elution at T3, T4, and T5. On the other hand, the peak elution from FT was at T2 followed by T1, and both were significantly higher (p < 0.05) than the elution at T4.

### Bis-EMA

**Fig 4:**
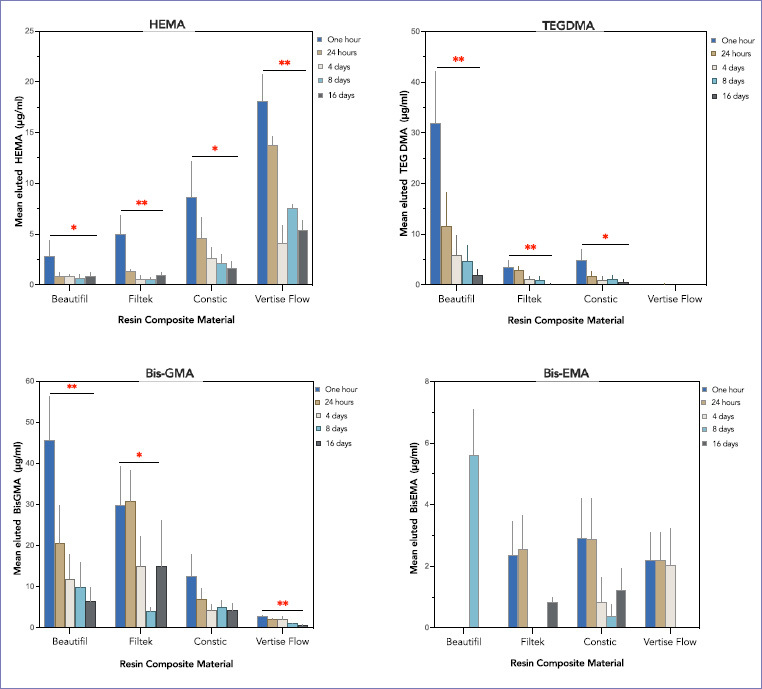
Mean concentration (µg/ml) of each residual monomer eluted from the tested resin composites for each immersion interval. Error bars represent standard error of the mean. Single asterisk denotes significant difference at p < 0.05, while double asterisks denote statistically significant difference at p < 0.0001.

All tested materials released bis-EMA monomer at different concentrations, and the observed differences were non-significant (p > 0.05). Constic released 31.6% of the total eluted bis-EMA, and it was detected at all time intervals (Fig 3). The elution from FT and VF was detected at only 3 intervals (FT: T1, T2 and T5) (VF: T1, T2, and T3), while the elution from BF was non-detectable except at T4 (Fig 4).

**Fig 5:**
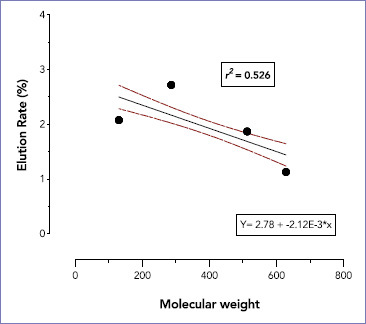
Scatter plot of the linear regression between the rate of monomer elution and monomer molecular weight (r^2^=0.526, p = 0.001).

## DISCUSSION

The current study investigated the elution behavior of self-adhesive resin composites using HPLC, specifically in relation to the release of four methacrylate monomers (ie, bis-GMA, bis-EMA, TEG-DMA, and HEMA) at five specified time intervals (total of 16 days), which was not addressed previously in the literature.

HPLC analytical methodology offers the advantage of controlling the separation process, particularly due to the solubility of monomers in the mobile phase. Identification and quantification of eluates is performed following calibration with standards solutions.^37,42^

It has been suggested that the morphology of the resin matrix and the internal distribution of the monomer within this matrix have a profound effect on the quantity of leached monomers.^49^ Additionally, different resin matrix sites contribute to the leaching process with different kinetics.^14^

In this study, we found that the monomer’s molecular weight was inversely correlated to its elution rate. Smaller monomers leached out at a faster rate than larger MW monomers. Molecular weight of the monomer accounted for 52.6% of the variations in the elution rate, a finding that is in line with another study.^1^

The chemical characteristics of the extraction solvent impacts the concentration and the rate of the released monomers.^27^ In the current study, we used 75wt% ethanol-water solution, which is considered the best composite resin solvent and food-simulating liquid that has been used in many studies on dental resin composites.^15^ Furthermore, it has been accepted by the US Food and Drug Administration (FDA) as a food simulator and aging accelerator.^38,49^ Since it is an aggressive medium, it can be considered as the worst-case scenario relative to the intraoral conditions. The intraoral fluids are within a range between water and the more aggressive organic solvents such as ethanol.^15^ Organic solvents can more easily penetrate and swell the organic matrix, increasing plasticization and sorption, thus promoting the release of unreacted monomers via expanding the spaces between polymer chains.^28,37,54^

In the present study, the extraction solution was refreshed at each analysis. Refreshing the extraction solvent is considered a clinically relevant approach (ie, simulating the continuous flow of saliva and oral fluids) in contrast to keeping the solvent unchanged throughout the experiment.^54^ This would also maintain a relatively constant pH over the experiment duration.^10^ Moreover, possible monomer degradation may occur, particularly in complex cross-linking monomers, if the extraction solvent is not refreshed.^29^ This approach must be taken into consideration when comparing the findings in the literature. Some studies report the cumulative elution concentration (ie, the extraction solution was not changed during the experiment), others, like this study, report the non-cumulative concentration of released monomers (ie, extraction solution was refreshed after each analysis).

In the current study, differences were observed in the elution rate of the monomers and their total mean elution concentration. The fastest monomers to elute from the tested materials were TEG-DMA followed by HEMA; both are co-monomers with relatively low molecular weight and higher mobility than larger molecules.^5^ On the other hand, bis-GMA exhibited the highest total mean concentration among the eluted monomers.

Bis-GMA is a common constituent monomer in all the tested materials, and it was detected at all time intervals. Despite being a structurally large molecule, bis-GMA exhibited the highest mean concentration of released monomer in the current study, which is in agreement with other studies.^25,39,43^ Although bis-GMA is highly viscous in nature, it exhibited a high water sorption capacity due to the presence of two pendant hydroxyl groups in its chemical structure.^41^ A possible explanation for the increased elution of this monomer is the fact that its high viscosity, rigidity (due to the presence of rigid aromatic nuclei), as well as high transition temperature impact its polymerization conversion (less double bond conversion), thus increasing the availability of unreacted monomers that leached out from the resin matrix especially in ethanol water solvent.^37,48^

Beautifil showed the highest vulnerability, with bis-GMA and TEG-DMA constituting the main eluates. A significantly higher concentration of bis-GMA was released, especially at T1 post-immersion. The elution curve of Beautifil showed a significantly higher initial elution peak followed by constant reduction over time. Beautifil is a giomer based on surface pre-reacted glass (S-PRG) fillers which contribute to the material’s fluoride-releasing mechanism. It has been reported that giomers have higher water absorption than do other RBCs.^13,21,26^ This has been attributed to the possible presence of S-PRG filler with its hydrogel layer containing fluoride complexes, which dissolve from the material through osmosis, resulting in higher water absorption, swelling and pressure. The presence of water molecules affect the material’s internal structure (eg, microvoids in the resin matrix, plasticization, or filler debonding), resulting in faster degradation or softening of the composite with subsequent release of unbound monomers.^13,26^

Filtek, which also released a significantly higher quantity of bis-GMA, showed a characteristic elution behavior that started with high elution at 1 h, then reached the peak at 24 h, followed by a sharp reduction over time until it increased slightly at day 16 (T5).

In contrast to the current findings, several studies reported lower total concentration of eluted bis-GMA.^5,24,52^ In a meta-analysis of elution studies,^54^ bis-GMA was reported to be released in lower concentrations compared to HEMA and TEG-DMA.

TEG-DMA is one of the most frequently used diluent hydrophilic monomers which reduce overall viscosity in composite materials and bonding resins.^12^ It is a main constituent of all tested materials in this study (except VF) according to the manufacturers’ data sheets. Due to its high mobility, it was eluted faster, with peak elution detected at 1 h in all materials. The high elution rate of TEG-DMA in 70% ethanol-water solution is consistent with other studies.^1,49^

The TEG-DMA-resin network is highly flexible and heterogeneous, with triethylene oxide spacers that increase the water sorption capacity of the monomer. This allows more water into the matrix micropores, easier monomer diffusion into extraction solvent, and subsequently faster decomposition.^10,11,41^

HEMA has the lowest molecular weight among the tested monomers in the current study. It was released from all tested materials, albeit in variable concentrations. The highest mean eluted concentration of HEMA (at one time point) detected in the current study was 18.1 ug/ml by VF at T1. HEMA continued to elute from all tested martials throughout the experiment duration, although the peak elution was detected at T1 for all materials.

Interestingly, HEMA was only mentioned as a main ingredient in the self-adhesive materials. It was the second constituent of Vertsie Flow (in the order it appears on the manufacturer’s data sheet), and it is the fourth component of Constic. It is assumed that the order in which the materials are listed is the order of predominance (highest to lowest concentration). HEMA was not mentioned as a component of Beautifil or Filtek (manufacturers’ information). Therefore, the detected eluates could be a reaction by-product of other monomers, or due to incomplete reporting of exact material composition by the manufacturers.^5,8,54^

Bis-EMA, which is the hydrophobic structural analog of bis-GMA, was the least eluted monomer in the current study, and it was the slowest to be released. This result is consistent with the findings from other studies.^1,8^ The low elution concentration of this monomer can be primarily attributed to its high molecular weight, which is greater than all the other tested monomers. Additionally, it has a stiff, central phenyl-ring core and a low water sorption capacity.^41,48^ In the current study, the only tested RBC that has bis-EMA listed as a main constituent is Filtek Z350 XT (manufacturer’s information). However, it was detected as an eluate from all tested materials. Interestingly, Constic was the only material that released bis-EMA at all time intervals, while Filtek release was limited to only T1, T2 and T5. It is possible that this monomer was added to the resin matrix of the other materials, but at a very low concentration (below 1%) and was therefore not mentioned in the MSDS.^28^ It might also be a decomposition by-product of other monomers, or part of manufacturing impurities.

Of all the tested RBCs, the lowest total monomer elution was detected for Vertise Flow. This can be attributed to its higher filler loading (by wt/vol%) compared to the other tested materials. It is known that the high weight percentage of the fillers is inversely related to water sorption,^10^ which would affect the leaching out of residual monomers. In other words, if high filler wt% reduces sorption, less solvent penetration into the resin matrix is anticipated, with less expansion of the resin network, and subsequently reduced leaching of monomers. However, this assumption needs further investigation.

The finding that peak elution of monomers occurred at 1 h (and within 24 h) post-immersion, corroborates the results of several other studies. Ferracane and Condon^17^ observed that monomer elution was completed within 24 h post-immersion in saliva and ethanol solvents. Pongprueksa et al^40^ reported a high initial release of monomers in the first week of the experiment, followed by a considerable decline in the second week. Another study on SDR flowable RBC reported that a significant majority of TEG-DMA was released within the first few hours after polymerization.^27^ Additionally, some studies observed a reduction in monomer elution after longer immersion periods (eg, 28 days) in various solvents.^38^

The biocompatibility of residual monomers identified in the current study has been extensively investigated in the literature, particularly the cytotoxic and genotoxic effects on various dental tissues and cell metabolic processes (eg, gingiva, pulp, and periodontal ligament).^18,20,46^ On the cellular level, resin monomers were found to reduce the levels of glutathione, which has a cellular protective effect against damage induced by reactive oxygen species, which contribute to cytotoxicity via apoptosis if a high level is reached.^46^ Moreover, the residual monomers may contribute to caries process via enhancing the bacterial proliferation.^20^

Intracellular redox metabolism is affected by TEG-DMA (concentration ≥2.5 mM) which also induces concentration-dependent DNA damage and other significant cyto- and genotoxic effects in human immortalized oral keratinocytes and other mammalian cell cultures.^46,55^ It has been also found to induce dose-dependent apoptosis in human pulp fibroblasts.^51^ Bis-GMA was found to be cytotoxic to human dental pulp cells (concentration > 0.075 mM).^7^ In cell cultures, HEMA was significantly less cytotoxic than other monomers.^18^

The quantity of released monomers in this study was below the toxic levels reported in the literature, particularly the comprehensive analysis by Geurtsen et al^18^ who determined the ED50 values for 35 dental monomers on permanent and primary dental fibroblast from gingiva, pulp and PDL.

It is important to acknowledge that the current analysis focused on the elution of the most common methacrylate monomers found in the four tested materials. Other studies have shown that other major monomers, co-monomers, filler particles, additives, and reaction by-products can be also extracted from polymerized RBC materials and detected in the elution analysis.^15,37^

When evaluating the amount of released monomers, it is important to consider the total exposed surface area of the tested specimen, as these factors were found to be significantly correlated.^25,54^ In the current study, the total surface area of the resin composite sample (33 mm^2^) is close to an estimated size of a box restoration, according to the method described by Van Landuyt et al.^54^ A higher concentration of eluted monomers is expected if the restoration involves teeth with larger surface areas or multiple RBC restorations. Additionally, the oral environment is affected by the enzymatic action of saliva, among other factors, which results in more elution of residual monomers compared to in-vitro detected concentrations.

## CONCLUSIONS

- No specific elution behavior can be attributed to self-adhesive RBCs. Elution of residual monomers is dependent on each material’s composition, resin matrix characteristics, and the monomer’s molecular weight.
- Elution rate was significantly dependent on the monomer’s molecular weight (p < 0.05).
- The peak elution of monomers occurs at 1 h post-immersion, after which the elution process significantly decelerates.
- The fastest monomer to elute was TEG-DMA, followed by HEMA, while the highest total mean concentration eluted was detected for bis-GMA.
- Beautifil Flow Plus released the highest mean concentration of monomers, particularly TEG-DMA and bis-GMA; while Vertis Flow released the highest mean concentration of HEMA.

## Clinical relevance:

The investigated resin composites showed an initial elution peak that continued over 16 days at a slower rate. This indicates that RBCs act as a chronic source of residual monomers in the oral environment.
